# Unraveling the Complexity of Vaginismus in Marital Relationship: A Case Series

**DOI:** 10.7759/cureus.73414

**Published:** 2024-11-10

**Authors:** Manoj K Sahoo, Harshita Biswas

**Affiliations:** 1 Psychiatry, Tata Main Hospital, Manipal Tata Medical College (Manipal Academy of Higher Education), Jamshedpur, IND; 2 Psychiatry, Tata Main Hospital, Jamshedpur, IND

**Keywords:** management, marital relationship, psychological factors, sexual dysfunction, vaginismus

## Abstract

This article presents a case series examining the interplay between marital dynamics and the management of vaginismus. Vaginismus is a spasm of the perivaginal muscles sufficient to prevent penile entry or make it uncomfortable. Given the multifactorial nature of vaginismus, with psychological and relational factors playing significant roles, understanding these dynamics is crucial for effective treatment. The series involves three females, each experiencing vaginismus linked to poor marital adjustment, uncertainty about the relationship, and emotional disconnect. Treatment included psychotherapy and medication for anxiety, leading to varying degrees of symptom relief and improvement in marital satisfaction. These cases highlight how crucial it is to investigate sexual pain disorders like vaginismus using a bio-psychosocial approach. The biological realm alone is not necessarily the only reliable indicator. Rather, interpersonal and psychological factors are important predictors of vaginismus. Evaluation of women’s psychosocial context may provide us with a deeper understanding of vaginismus and its impact on both women and their partners.

## Introduction

Sexual dysfunction in women encompasses a wide range of persistent problems related to sexual desire, arousal, orgasm, and pain during intercourse, which have a significant impact on emotional and relational well-being. These dysfunctions can stem from various factors, including hormonal imbalances, medical conditions, medications, and psychological issues such as stress, anxiety, or past trauma. According to the International Classification of Diseases, 10th Revision (ICD-10) [1], non-organic vaginismus is a spasm of the perivaginal muscles sufficient to prevent penile entry or make it uncomfortable. Vaginismus is multifactorial, with potential psychological, cultural, and physiological underpinnings, requiring a multidisciplinary approach to diagnosis and treatment [2,3].

Women suffering from sexual dysfunction vary globally; it affects approximately 40-45% of women worldwide. Vaginismus affects around 5-17% of women, depending on the population studied [4]. In the Indian population, research on sexual dysfunction is limited, but available studies suggest a prevalence of 20-30%, with vaginismus being reported less frequently [5,6].

Marital relationships significantly influence a couple's sexual life, as emotional quality and interpersonal dynamics impact sexual functioning. Factors like communication, trust, intimacy, and mutual satisfaction are crucial. Marital strain, unresolved conflicts, or power imbalances can lead to sexual dysfunctions like vaginismus, highlighting the importance of addressing relational factors in treatment [3,7].

In the literature review, studies have indicated that lack of intimacy with a spouse, sexual satisfaction, and marital satisfaction were significantly associated with genito pelvic pain/penetration disorder (GPPPD) [8]. A study by Warden and Ogden (1994) [9] revealed that one of the causes of vaginismus in women is the fear of intimacy with the husband, which prevents close relationships and intercourse. The study also highlighted how relationship ambiguity affects sexual dysfunctions, especially vaginismus. According to studies, this disorder may be related to problems with power, trust, and the nature of relationships [9]. Probably, such relationship-associated anxieties can transform into physical symptoms, intensifying the fear of pain and making attempts at intercourse painful.

Despite its universal prevalence, vaginismus remains under-researched [10]. Nga (2010) [11] highlighted that societies that suppress female sexuality and place high importance on female virginity have higher incidences of sexual dysfunction comprising vaginismus due to the clash between intrapersonal, interpersonal, and cultural aspects. It is possible that women become sexually insensitive as a result of conflicts between their own sexual impulses and procreation and anti-sexual norms established by their families and cultures. This indicates that vaginismus needs to be examined at the intrapersonal and cultural levels in order to be completely understood. The role of interpersonal relationships in vaginismus is the one that has been the least explored in the literature. Therefore, before addressing vaginismus, couple therapy should address unsolved relationship issues. Compared to other components like physical, family, prior trauma, and social, there is a dearth of studies on interpersonal dynamics and their association with vaginismus, and no studies have directly addressed the lack of certainty about staying in a marriage [10].

We present the scenarios of three patients presenting with vaginismus resulting from poor marital adjustment and insecure marital relationships. This highlights the intricate connection between psychological and interpersonal factors in vaginismus. Understanding the dynamic can help in more effective therapeutic intervention, both for treating vaginismus as well as addressing marital conflicts.

## Case presentation

Case 1

A 25-year-old female graduate, married for four years, visited the gynecology outpatient department for evaluation and management of primary infertility. Routine investigations, hormonal profile, and necessary detailed infertility work-up of the patient and her husband by the gynecologist and urologist revealed no abnormality. A vaginal examination was not possible because of the spasm of the lower third of the vagina, and it was not associated with any demonstrable local pathology. Subsequent psychiatric assessment revealed that her marriage was not consummated because of fear of pain during penile penetration. She had tried penetration with alcohol, xylocaine jelly, and drugs, and she had undergone minor surgery to widen her vaginal introitus without any benefit. On exploration, it was revealed that the patient did not have a good interpersonal relationship with her husband, and she wanted to consummate the marriage only to bear a child; she also had phobias of blood and injections. Over the course of time, she was successfully treated through behavior therapy for her vaginismus and other phobias, as she did not want to address her marital issues. The patient was asked to make a hierarchy list of anxiety-provoking situations surrounding her fears. The Subjective Unit of Distress (SUDS) was assigned to each situation, and exposure was started with the lowest SUDS score situation. The exposure included imaginal to in vivo exposure of progressive SUDS. The exposure therapy was carried out under the cover of relaxation therapy, supervised by the therapist and co-therapist (husband). Regular sessions lasting for 30-45 minutes were carried out. It took four months and 14 sessions to complete all the hierarchy list situations and simultaneously required medication for her anxiety. The patient was accompanied by her husband for the sessions, and he was involved in the treatment process. She was prescribed tablet escitalopram 10 mg/day and tablet clonazepam 0.25 mg/day for anxiety symptoms. She continued her medications until pregnancy. She conceived and gave birth to a healthy male child. She dropped from the follow-up and visited after two years with complaints of depression and anxiety symptoms. On exploration, we found that just after the birth of her child, she continued to have intense fear and did not allow for physical intercourse but continued to have other forms of sexual pleasure with her husband. Additionally, she disclosed an extramarital affair and reported a stronger emotional and physical connection with her new partner, noting persistent dissatisfaction in her marriage. However, she never engaged in vaginal intercourse and resorted to other forms of sexual pleasure.

Treatment at this stage included desvenlafaxine (100 mg/day) for depression and cognitive-behavioral therapy (CBT) over 12 sessions, focusing on identifying cognitive distortions and establishing adaptive coping mechanisms. Although her interpersonal relationship with her husband remained strained, she reported psychological relief and symptom management in her new relationship context.

Case 2

A 24-year-old female, married for six months, came along with her husband with complaints of not being able to engage in intercourse due to fear of pain and injury. The patient’s husband reported that the patient agrees and engages in anal penetration and does not report any distress to it. On detailed exploration, it was found that the patient was not ready for marriage and wanted to pursue her studies and work for some time, which ultimately did not happen. Also, her husband was eight years older than her and wanted to start a family as soon as possible. The patient was not psychologically ready to conceive and take such a huge responsibility with only six months into marriage. She was subsequently advised for therapy to address her issues in marriage and sexual difficulties and medications (capsule fluoxetine 20 mg/day) for her anxiety symptoms. The patient came along with her husband for the 10 sessions of therapy. We focused on marriage-related issues, addressing the acceptance of the relationship, emotional intimacy, allowing a safe place for the patient to share her feelings, and improving the communication pattern. Subsequently, we moved to treating her vaginismus, where, again, the husband was part of the therapy. Through systematic desensitization (in vivo, imaginal, or both), together with the use of relaxation techniques and gradually inserting a dilator to insert the finger into the vagina to reduce the anxiety and fear associated with vaginal intercourse. The session went on for two months and eight sessions. She was continued on capsule fluoxetine 20 mg/day. The patient showed improvement in terms of sexual functioning, and the couple has decided not to start a family for a couple of years.

Case 3

A 35-year-old female, postgraduate, working in a multinational company, married for four months, came along with her husband with complaints of being unable to engage in intercourse due to fear of pain and injury. In a detailed interview, various aspects of her marital and sexual history were explored. The patient was engaging in foreplay, but she could not engage in vaginal intercourse. While discussing her marital life, it was discovered that she had an arranged marriage, and the patient’s husband was in a relatively low job profile as compared to her and earned less salary than her. As the patient was over 30 years of age, her parents had difficulty in finding an alliance; hence, even though he was on a lower profile, they had convinced her to the marriage. After marriage, the patient started feeling unsure about the relationship as she found her husband intellectually and financially weaker than her. When these aspects were discussed, the patient was reluctant to accept the psychological reasons and expected that she would be prescribed medications. The patient came for three sessions of therapy along with her spouse, which focused on her insecurities and marital life. The three sessions of the therapy, each lasting for 45-60 minutes, focused on her thoughts and behaviors that were impacting their relationship, helping her identify the unrealistic standards she had developed toward relationships and gradually address her thoughts related to physical intimacy. The patient subsequently dropped out after three sessions.

## Discussion

The cases presented demonstrate that vaginismus does not exist in isolation but is intertwined with the patient’s emotional and relational context. In cases of relationship uncertainty, women may develop a complex response to intimacy, where the desire to maintain the relationship conflicts with fear or discomfort around intercourse. This tension is reflected in the progression of symptoms and the emotional toll it takes on both partners. Studies support that women experiencing vaginismus are more likely to report feeling disconnected or dissatisfied with their partner, suggesting that relationship quality plays a central role in either exacerbating or alleviating symptoms [7].

In the first case, the patient’s motivation to consummate the marriage was only to bear a child. She again developed symptoms of vaginismus soon after childbirth. Similar findings also have been described in previous literature [12]. In the second and third cases, the emotional connection was missing, which resulted in disturbed sexual intimacy. Research also suggests that marriages with frequent arguments, mistrust, or emotional distance result in stress and anxiety, which can manifest as physical symptoms [13]. Also, difficulty in the emotional relationship with the partners can predict emotional distress during the sexual encounter [14].

As mentioned in all the cases, the partners or spouses play a crucial role in the psychological treatment of vaginismus. By involving partners in the therapeutic process, clinicians can help couples improve communication, build trust, and create a supportive environment for addressing intimacy concerns. The complex dynamics of the relationship, which is typically unconscious, could be explained in the experiential aspects of therapy, leading to personal and relational insights that can be catalysts for change [15].

Women in strained marriages may feel pressured to engage in sexual activity despite emotional distress, which can intensify their fear of penetration, ultimately reinforcing the cycle of pain and avoidance. This cycle often leads to deeper relational problems, creating a vicious loop between poor marital life and sexual dysfunction. The choice of partner plays a significant role in sexual dysfunction, including vaginismus, as relationship satisfaction and partner behavior directly influence sexual health [3].

In terms of clinical implications, these cases emphasize the importance of using a bio-psychosocial approach when investigating sexual pain disorder. Only the biological domain cannot always be set as a strong predictor. Instead, psychological and interpersonal variables play a prominent predictor of vaginismus. Assessment of women’s psychosocial context may provide us with a deeper knowledge of vaginismus and its impact on both women and their partners [16]. The need for a holistic approach to treating vaginismus that includes relationship/marital counseling alongside pharmacological treatment is essential. Addressing marital discord, improving communication between partners, and fostering emotional closeness are crucial steps in mending the marital relationship and treating sexual dysfunction.

## Conclusions

This case series underscores the complex relationship between marital uncertainty and primary vaginismus, highlighting how emotional and relational conflicts can manifest as physical symptoms that disrupt sexual functioning. These cases illustrate that vaginismus cannot solely be viewed as a physiological issue; rather, it is deeply rooted in the interpersonal and psychological landscape of intimate relationships. The findings emphasize the need to consider marital and relational factors when diagnosing and understanding vaginismus, particularly in cultural contexts where open discussions of sexuality remain limited. Recognizing relationship dynamics can aid in developing more comprehensive assessments, guiding clinicians toward interventions that address not only the individual’s symptoms but also the broader relational context. Further research is necessary to explore these dynamics in diverse populations. A deeper understanding of relational influences on vaginismus may offer new perspectives for addressing sexual dysfunctions in culturally sensitive ways.
